# Retrospective case-control analysis of the infectious complications of retained broken needles in people who inject drugs

**DOI:** 10.1097/MD.0000000000041511

**Published:** 2025-02-21

**Authors:** Alex Sandberg, Shalom Mammen, Angela Udongwo, Daohai Yu, Xiaoning Lu, Ryan Graham, Gary Cohen, Hillel Maresky

**Affiliations:** a Lewis Katz School of Medicine at Temple University, Philadelphia, PA; b Department of Biomedical Education and Data Science, Center for Biostatistics & Epidemiology, Lewis Katz School of Medicine at Temple University, Philadelphia, PA; c Department of Radiology, Temple University Hospital, Philadelphia, PA.

**Keywords:** retained broken needle, people who inject drugs, infectious complications, incidence, underreported, imaging challenges, incidental

## Abstract

Retained broken needles (RBNs) in patients is a potentially underreported complication of intravenous drug use (IVDU) in people who inject drugs. Identification of RBNs poses a challenge for radiologists and clinicians due to subtle appearance on imaging and the complexities of patient presentations. A single-center case-control study was performed between January 1, 2021, and December 31, 2021. The cases included all patients with a history of IVDU who presented to the emergency department (ED) with RBNs. Data collected on the study population included RBN location and size, complications, and imaging modality. A control group of 2:1 matched on age, gender, and race/ethnicity with the cases was generated from patients with a history of IVDU from the same time period who had no RBNs. A total of 3920 total patients presented to ED with a history of IVDU and 70 patients were found to have RBN (1.8%). RBNs were most observed in the foot/ankle (32.9%) forearm (18.6%), and neck (17.1%). RBNs were found to be incidental in 82.9% (58 out of 70) of patients. Radiography located needle fragments (55.7%) slightly more often than CT (44.3%). Overall, among all the cases and controls combined, 33.8% had positive blood cultures. In the group of patients with a RBN, 47.1% presented with positive bacterial blood cultures during the hospital admission, whereas the group without an RBN showed 27.1% (*P* = .004). The RBN group showed significantly higher rates of abscess (72.9% vs 48.6%), cellulitis (70.0% vs 46.4%), and osteomyelitis (37.1% vs 13.6%) than the non-RBN group (all *P* ≤ .001). This study suggests that RBNs are likely underreported and patients with RBNs are at an increased risk of infectious complications. Increasing the awareness and refining our understanding of RBNs is crucial to mitigating these complications.

## 1. Introduction

Hypodermic needles used by people who inject drugs (PWID) can experience structural weakening due to repeated use, increasing the likelihood of breakage within the damaged soft tissues.^[1,2]^ In some cases, the needle may break off at the hub, embedding into the underlying tissue. This can make retrieval difficult and lead to retained broken needles (RBNs). One survey study conducted among Bristol County prison inmates who engage in intravenous drug use (IVDU) revealed that 20% of participants reported experiencing a broken needle in the past.^[2]^ While the incidence of RBNs in the PWID population remains a mystery, it is likely underreported given the increase in injection drug use nationwide, the lack of standardized reporting, and the challenge to identify RBNs.

Alongside the well-documented complications associated with IVDU, the presence of RBN fragments introduces an additional layer of morbidity including injection site infections, needle stick injuries to healthcare workers, and, in severe cases, intravascular needle embolism.^[3–5]^ Comprehensive research has not yet been conducted on the frequency of RBNs and their associated clinical outcomes. The primary objective of this study is to investigate the prevalence of RBNs in PWID as well as to quantify if patients with RBNs are at higher risk of infectious complications. The secondary aim is to describe the initial clinical management and anatomic distribution of RBNs in patients.

## 2. Materials and methods

### 2.1. Study design and patient populations

A comprehensive retrospective review of electronic medical records (EMR) was conducted to identify all patients with a history of IVDU presenting to the Temple University Hospital emergency department (ED) with RBNs being identified in 2021. Demographic information including age, sex, and race was documented. Detailed information regarding the date and time of ED presentation, primary clinical presentation, and imaging used to identify RBN were also documented. All imaging reports were verified by attending radiologists to confirm the presence of RBNs. Lastly, complications associated with RBNs including the presence of an abscess, osteomyelitis, cellulitis, and blood culture results during admission were recorded.

A control group was generated through a data extraction from the EMR of all patients with a history of IVDU presenting to the ED in 2021. Patients in the control group lacked radiographically confirmed RBNs. This control cohort underwent a 2:1 randomized matching individuals based on age, sex, and race to the RBN group. The identical data parameters were recorded as previously outlined. The study’s protocol was approved by the Temple University Institutional Review Board (IRB Protocol # 29951-0004, November 2022).

### 2.2. Statistical analysis

Data were expressed as counts and percentages for categorical variables and median (range or quartile range) for continuous variables. Comparisons between the cases and controls were performed using Wilcoxon rank sum test for a continuous variable (e.g., age) and the Fisher Exact mid-*P* value or chi-square test for a categorical variable (e.g., sex or race). Raw/unadjusted odds ratios (95% CIs) were also reported to explore the associations between having a RBN and each of the underlying complications of interest. Furthermore, a stepwise multivariable logistic regression was performed to select the best subset of the underlying complications that was associated with having a RBN independent of one another. Matching variables (i.e., age, sex, and race) between the cases and controls were forced into the multivariable logistic regression model. Adjusted odds ratios (95% CIs) were calculated along with 95% confidence intervals based on the final multivariable logistic model. P values <.05 were considered statistically significant. SAS version 9.4 (SAS Institute Inc., Cary) was used for all the data analyses.

## 3. Results

### 3.1. Initial evaluation of RBN

**Figure 1.**
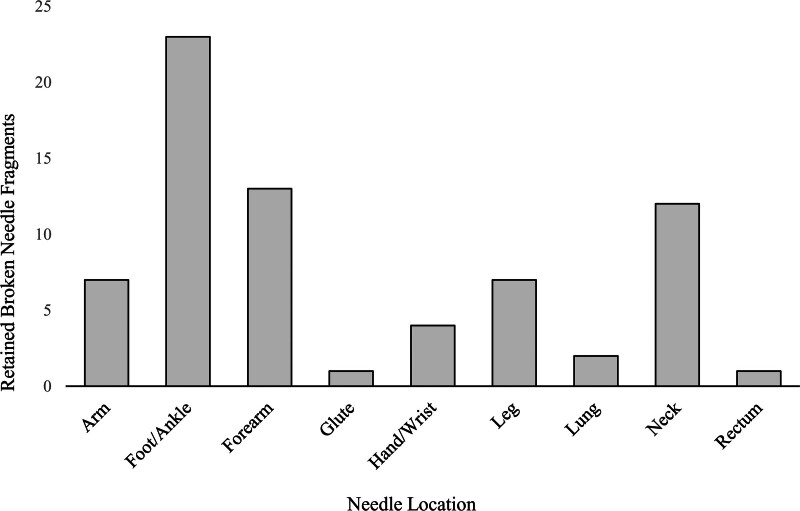
Anatomic location of retained broken needles discovered in 70 patients presenting to the emergency department.

**Figure 2.**
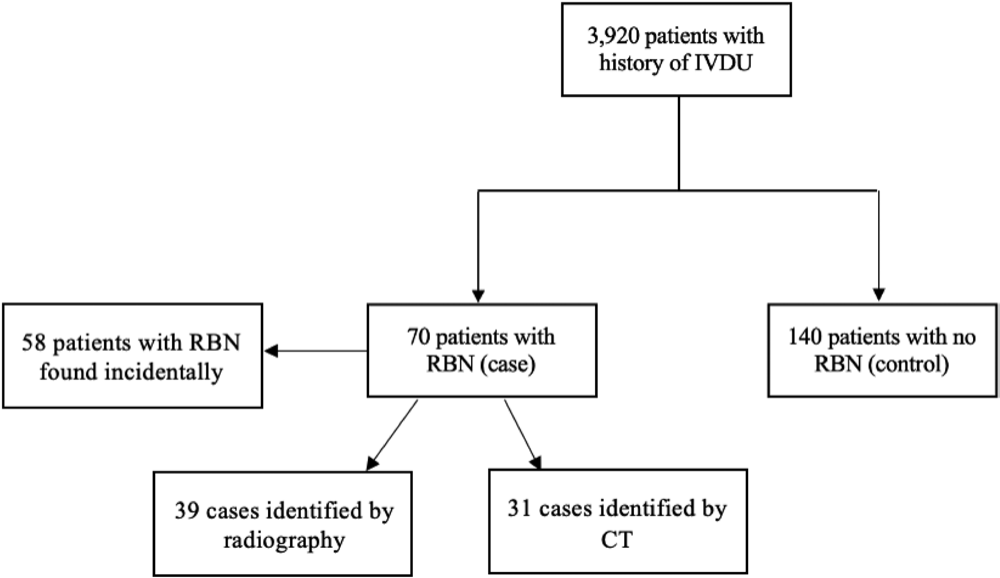
Flow chart representing study populations. CT = computed tomography, IVDU = intravenous drug use, RBN = retained broken needle.

A total of 3920 patients presented to ED with a history of IVDU and among them, 70 patients were found to have RBN (1.8%). From this same patient cohort, 140 individuals without RBNs were designated as the non-RBN control group. The most frequently observed RBN locations was the foot/ankle (32.9%), forearm (18.6%), and neck (17.1%). These values are graphically represented in Figure 1. Incidental findings, defined as the presence of an RBN that was not reported by the patient, accounted for 82.9% of cases. Radiography (55.7%) was slightly more utilized than CT (44.3%) in first identifying RBNs. Notably, 45% RBNs were incidentally discovered by radiography and 37% were incidentally identified by CT. A flow chart detailing the study groups is presented in Figure 2.

### 3.2. The clinical complications between RBN versus non-RBN groups

Overall, among all the cases and controls combined, 33.8% (71 out of 210) had positive blood cultures, indicating a systemic infection. In the RBN group, 47.1% (33 out of 70) presented with positive bacterial blood cultures during the hospital admission, compared to the control group without an RBN that showed only 27.1% (38 out of 140). The RBN group exhibited significantly higher rates of systemic infection than the non-RBN group (*P* = .004). Among the identified pathogens, staphylococcus species was the most prevalent in both groups combined, accounting for 53.5% (38 out of 71) of all positive cultures. Methicillin-resistant Staphylococcus aureus (MRSA) was responsible for 33.8% (24 out of 71) of all positive cultures between the 2 groups. The RBN group showed slightly higher rates (15.7%) of MRSA infection compared to the non-RBN group (9.3%), but it was not statistically significant (*P* = .17). Streptococcus species was the culprit in 33.8% (24 out of 71) of all positive blood cultures in the 2 groups. Demographic and clinical complications in each study group are recorded in Table 1.

**Table 1:** Demographics and raw/unadjusted odds ratios (95% CIs) of having a broken needle with each of the complications of interest.

Variable	Total (n = 210)	Case (n = 70)	Control (n = 140)	Unadjusted *P* value	Unadjusted odds ratio (95% CI)
Age median (range)*	39.0 (17.0, 79.0)	39.0 (17.0, 79.0)	38.0 (19.0, 76.0)		
Sex (%)					
F	78 (37.1)	26 (37.1)	52 (37.1)		
M	132 (62.9)	44 (62.9)	88 (62.9)		
Race (%)*					
White	114 (55.1)	38 (55.1)	76 (55.1)		
Black	45 (21.7)	15 (21.7)	30 (21.7)		
Hispanic	48 (23.2)	16 (23.2)	32 (23.2)		
Incidental (%)					
Yes		58 (82.9%)			
No		12 (17.1%)			
Modality (%)					
XR		39 (55.7%)			
CT		31 (44.3%)			
Abscess (%)				<.001	2.84 (1.53–5.30)
Yes	119 (56.7)	51 (72.9)	68 (48.6)		
No	91 (43.3)	19 (27.1)	72 (51.4)		
Osteomyelitis (%)				<.001	3.76 (1.90–7.46)
Yes	45 (21.4)	26 (37.1)	19 (13.6)		
No	165 (78.6)	44 (62.9)	121 (86.4)		
Cellulitis (%)				.001	2.69 (1.46–4.95)
Yes	114 (54.3)	49 (70.0)	65 (46.4)		
No	96 (45.7)	21 (30.0)	75 (53.6)		
Blood culture (%)				.004	2.39 (1.31–4.36)
Yes	71 (33.8)	33 (47.1)	38 (27.1)		
No	139 (66.2)	37 (52.9)	102 (72.9)		
Staph (%)				.61	1.21 (0.58–2.51)
Yes	38 (18.1)	14 (20.0)	24 (17.1)		
No	172 (81.9)	56 (80.0)	116 (82.9)		
MRSA (%)				.17	1.82 (0.77–4.31)
Yes	24 (11.4)	11 (15.7)	13 (9.3)		
No	186 (88.6)	59 (84.3)	127 (90.7)		
Strep (%)				1.00	1.00 (0.41–2.46)
Yes	24 (11.4)	8 (11.4)	16 (11.4)		
No	186 (88.6)	62 (88.6)	124 (88.6)		

CI = confidence interval, CT = computed tomography, MRSA = methicillin-resistant staphylococcus aureus, SD = standard deviation, Staph = Staphylococcus species, Strep = Streptococcus species, XR = X-ray.

* These variables had the following numbers of missing values, respectively: age = 3; race = 3.

The RBN group showed statistically significantly higher rates of abscess, cellulitis, and osteomyelitis than the non-RBN group in univariable analyses (all *P* ≤ .001). 72.9% (51 out of 70) of patients in the RBN group were found to have abscess as opposed to 48.6% (68 out of 140) in the group without an RBN (*P* = .001). In the RBN group, 70.0% (49 out of 70) had cellulitis, whereas in the group without an RBN 46.4% (65 out of 140) were found to have cellulitis (*P* = .001). Osteomyelitis was found in 37.1% (26 out of 70) of the RBN group compared to only 13.6% (19 out of 140) in the non-RBN group (*P* < .001). After adjusting for all the 3 matching variables (i.e., age, sex, and race) osteomyelitis (*P* *=* .004) and abscess (*P* = .01) were the best subset of the complications associated with having an RBN (Table 2). Blood culture and cellulitis were not found to be significant in this stepwise multivariable logistic regression analysis.

**Table 2:** Adjusted odds ratio (95% CIs) of having a broken needle with the best subset of complications via a stepwise multivariable logistic regression (n = 207*).

Variable	β	SE	*P* value	Adjusted† OR (95% CI)
Abscess	0.884	0.355	.0128	2.42 (1.21–4.85)
Osteomyeltis	1.064	0.368	.0038	2.90 (1.41–5.96)

CI = confidence interval, OR = odds ratio, SE = standard error.

* n = 207 due to the missing data in age and race.

† Further adjusted for age, sex, and race.

## 4. Discussion

The opioid crisis continues to be one of the deadliest and most pressing healthcare issues facing the United States. According to a recent public health study by Bradley et al,^[6]^ it is estimated that 1.5% of American Adults injected drugs in 2018. Among the regions profoundly affected by this epidemic, North Philadelphia stands out as a significant hotspot. A record-breaking 1276 people died in Philadelphia of unintentional opioid overdose in 2021, which was the time period and location of this study.^[7]^

While the cohort of this study is limited to 70 patients, to date, this is the largest study investigating the incidence and rates of infectious complications in patients with RBNs. We report an incidence rate of RBN 1.8% in PWID. Given the notable rise in intravenous drug use in the nation, there are likely hundreds of thousands of people living with a RBN in their body. This statistic is likely an underestimation of the incidence of RBNs. The theorized underreporting of RBNs is multifactorial. Patients may be unaware of the presence of an RBN or reluctant to disclose its existence to healthcare providers. This was the case in 82.8% of individuals in this study, and there have been multiple case reports citing similar clinical presentations.^[8–10]^

It is well established that patients’ lack of disclosure may be an expression of societal stigma associated with PWID.^[11,12]^ Patients may feel shame and embarrassed when seeking medical care and expect to be blamed for their health issues.^[13]^ The negative stigma held against PWID likely contributes to the distrust of patients with drug-related complications and delays medical care. Additionally, PWID tend to have more complicated clinical presentations with higher rates of infectious comorbidities.^[14]^ As a result, the presence of a RBN may not be an immediate concern leading to decreased reporting and vigilance among clinicians.

**Figure 3.**
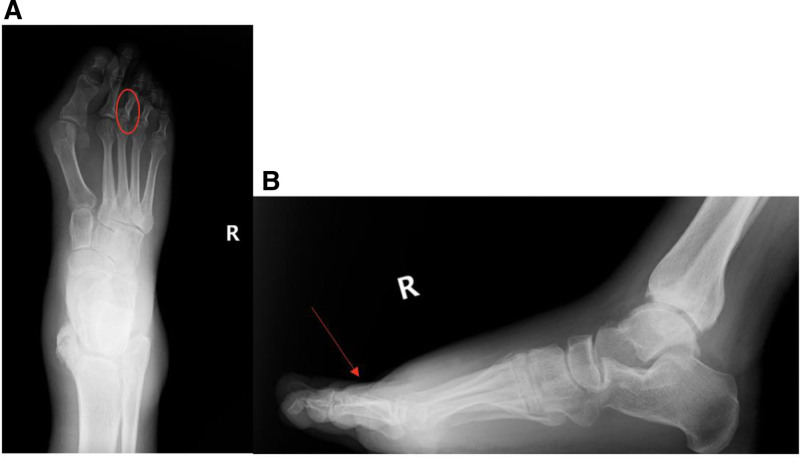
37-year-old male with a right foot abscess and a gradual onset of severe pain located on the medial instep of the right foot. Patient had X-ray foot right anterior-posterior and lateral imaging completed same day of admission. A 10 mm retained broken needle is positioned on the dorsal aspect of the proximal phalanx of the right third digit. The retained broken needle exhibits slightly enhanced visibility in the lateral film (B) compared the anterior-posterior film (A) in the setting of soft tissue inflammation.

The subtle nature of a needle fragment on imaging may be challenging to identify by radiologists who are not accustomed to seeing RBNs on routine imaging. Parameters such as small fragment size, superimposition on adjacent structures, shortcomings of plane of section on imaging, and limited familiarity with the appearance of RBNs contribute to the difficulties faced by radiologists in detecting these fragments as shown in Figure 3.^[3,8,15]^ Furthermore, the lack of a standardized protocol for reporting and managing RBNs among healthcare professionals may further contribute to the overall inconsistency in the reporting.

While there is limited comprehensive data regarding the rates of infectious complications in PWID, blood, skin, soft tissue and bone infections have been well documented.^[16]^ Complications including abscess, cellulitis and most severely osteomyelitis if left untreated can lead to more disastrous outcomes for patients.^[17]^ In this study, the finding that 47.1% of patients with RBN fragments presented with positive bacterial blood cultures is nearly double the rate found in the matched control population. Staphylococcus was the most common organism found in cultures, which is a well-documented culprit in PWID. Additionally, compared to the non-RBN control group, the presence of abscess and cellulitis showed an approximate 50% increase in the RBN group whereas osteomyelitis were nearly tripled. Notably, after further adjustment for matching variables, the more severe complications of osteomyelitis and abscess formation were most associated with the presence of RBNs. These findings substantiate the hypothesis that RBNs can serve as a nidus for bacterial proliferation resulting in infectious complications.^[3]^ Another possible explanation may be that individuals with RBNs are likely chronic drug users, potentially placing them at a higher risk for negative health outcomes. While this assertion serves as plausible justification for the findings in this study, further research is required to support this association.

The substantial increase in blood, skin, soft tissue and bone infections underscores the importance of timely identification and management among caregivers serving PWID. Proactive management may include standardized protocol with RBN appropriate antibiotics to mitigate complications upon patient presentation. Promptly identifying RBNs may further help prevent complications related to infection and others more rare consequences such as needle embolism.

Imaging remains a key factor in the diagnosis of RBN fragments. Recent studies have outlined a multi-step protocol based on mechanism of retention, but it has yet to be studied in clinical practice.^[15]^ In this study, most patients (83.1%) presented to the ED with trauma injury or soft tissue infections without knowledge of an RBN. These patients underwent imaging via radiograph or CT, eventually to discover an RBN. Although radiography was utilized slightly more often than CT (55.7% vs 44.3%) within the RBN group, this is presumably to the nature of the injury. Both conventional radiograph and CT are sensitive to radiopaque needle fragments, yet RBNs from IVDU may be challenging to identify. Radiography is often used as first line for many of the patients in this study as part of the emergency department protocol. CT may be followed if there remains a high suspicion for an RBN. In many cases, RBNs may be first discovered incidentally on CT due the enhanced visualization of the imaging modality.

Lower extremity imaging with positive RBN identification represents a significant subset (31.0%) of participants within this study. Existing research indicates that initial injection sites for most individuals engaged in injecting drug use are typically located in the arm.^[18]^ However, over subsequent years of continued drug use, these individuals tend to transition to alternative locations for venous access, including the foot, ankle, gluteal region, leg/groin area, neck, rectum, and hand. While limited investigations have focused on secondary injection sites for intravenous drug use, there is greater risk for complications of injection.^[19]^

The utilization of secondary injection sites is associated with infectious and vascular complications, such as direct limb ischemia and pseudoaneurysm formation.^[20]^ It is challenging for a person to see where they are injecting, which likely increasing the risk of injecting in undesirable locations.^[19]^ Notably, sites such as the neck and groin pose heightened risks due to the potential for detrimental effects on neighboring organs or vascular structures.^[8]^ The heightened frequency and risk of complications from injections in secondary sites should make caregivers more vigilant and adapt their management approach accordingly.

## 5. Limitations

Limitations in this study include the retrospective nature of the study, potential biases associated with data collection, and the limited size of the cohort. It is important to note the limited generalizability of the study. The characteristics and demographics of the study population, as well as the healthcare practices and resources available in the study setting, may differ from other locations. The reliance on electronic medical records may have resulted in missing or incomplete data for some patients that may have impacted the results of this study. Additionally, since there lacks standardized coding for RBNs in the medical record, the RBN case group was obtained through generalized searches of key words in phrases. There are likely patients in this group missing leading to a lower reported incidence. Years of drug use was omitted in this study due to lack of consistency in the medical records, which may be a confounder for many reported results.

## 6. Conclusion

This retrospective, case-control study suggests that RBNs are likely underreported in PWID. These patients are at an increased risk of infectious complications compared to those in the non-RBN PWID population. Therefore, prompt radiological identification of RBNs and appropriate communication to the clinical team may be critical.

To optimize the detection of occult RBNs, it is crucial to enhance radiologists’ knowledge of their appearances and increase awareness of RBNs. RBNs are likely to be found in secondary injection sites and it should be known that these patients are at particularly high risk. Further research and the development of wider clinical protocols may serve to further enhance the overall management of RBNs in PWID. By refining our understanding of RBN related complications and optimizing treatment strategies, we may begin to improve the care of individuals affected by this unique clinical challenge.

## Author contributions

**Conceptualization:** Alex Sandberg, Angela Udongwo, Ryan Graham, Hillel Maresky.

**Data curation:** Alex Sandberg, Shalom Mammen.

**Formal analysis:** Alex Sandberg, Daohai Yu, Xiaoning Lu.

**Investigation:** Alex Sandberg, Shalom Mammen, Angela Udongwo.

**Methodology:** Alex Sandberg.

**Supervision:** Hillel Maresky, Gary Cohen.

**Visualization:** Alex Sandberg, Shalom Mammen.

**Writing – original draft:** Alex Sandberg.

**Writing – review & editing:** Alex Sandberg, Shalom Mammen, Angela Udongwo, Daohai Yu, Xiaoning Lu, Ryan Graham, Gary Cohen, Hillel Maresky.
